# Antioxidative Effects of Standardized *Aronia melanocarpa* Extract on Reproductive and Metabolic Disturbances in a Rat Model of Polycystic Ovary Syndrome

**DOI:** 10.3390/antiox11061099

**Published:** 2022-05-31

**Authors:** Jovan Rudic, Vladimir Jakovljevic, Nikola Jovic, Maja Nikolic, Jasmina Sretenovic, Slobodanka Mitrovic, Sergey Bolevich, Stefani Bolevich, Miroslav Mitrovic, Sasa Raicevic, Kristina Andric, Andjela Dimkic Milenkovic, Dejana Rakic, Jovana Joksimovic Jovic

**Affiliations:** 1 Clinic for Gynecology and Obstetrics Narodni Front, 11000 Belgrade, Serbia; jovanrudic1@gmail.com; 2 Department of Physiology, Faculty of Medical Sciences, University of Kragujevac, Svetozara Markovica 69, 34000 Kragujevac, Serbia; drvladakgbg@yahoo.com (V.J.); majanikolickg90@gmail.com (M.N.); drj.sretenovic@gmail.com (J.S.); 3 Department of Human Pathology, I.M. Sechenov First Moscow State Medical University, 119991 Moscow, Russia; bolevich2011@yandex.ru; 4 Department of Gynecology and Obstetrics, Faculty of Medical Sciences, University of Kragujevac, Svetozara Markovica 69, 34000 Kragujevac, Serbia; docctorny@gmail.com (N.J.); dejavulovic@gmail.com (D.R.); 5 University Clinical Center Kragujevac, Zmaj Jovina 30, 34000 Kragujevac, Serbia; smitrovic@medf.kg.ac.rs (S.M.); kristinajoksimovic16.2016@gmail.com (K.A.); 6 Department of Pathology, Faculty of Medical Sciences, University of Kragujevac, Svetozara Markovica 69, 34000 Kragujevac, Serbia; 7 Department of Pathophysiology, I.M. Sechenov First Moscow State Medical University, 119991 Moscow, Russia; alistra555@mail.ru; 8 Department of Pharmacology, I.M. Sechenov First Moscow State Medical University, 119991 Moscow, Russia; 9 Pharmanova, St. Miloja Djaka No 1, 11010 Belgrade, Serbia; miroslav.mitrovic@pharmanova.com; 10 Medical Faculty, University of Montenegro, 81000 Podgorica, Montenegro; sasar@doctor.com; 11 Department of Dermatovenerology, Faculty of Medical Sciences, University of Kragujevac, Svetozara Markovica 69, 34000 Kragujevac, Serbia; 12 Clinic for Burns, Plastic and Reconstructive Surgery, University Clinical Center of Serbia, Zvecanska 9, 11000 Belgrade, Serbia; adimkic@yahoo.com

**Keywords:** polycystic ovary syndrome, estrus, metformin, *Aronia melanocarpa*, dehydroepiandrosterone, testosterone, oxidative stress, antioxidants, animal model, rats

## Abstract

Polycystic ovary syndrome (PCOS) represents the most common endocrinopathy among childbearing-age women, with oxidative stress (OS) underlying its etiopathogenesis. Metformin (MET) represents a frequently used agent in PCOS. However, weak results encourage alternative treatments. We aimed to investigate isolated and synergistic effects of Standardized *Aronia melanocarpa* extract (SEA) and MET for alleviating reproductive and metabolic PCOS abnormalities. PCOS induction was followed by 28-day treatment with MET, SAE, or MET + SEA. Bodyweight (BW), cyclicity, histological, and ultrasonographical ovarian analyses were performed. Hormonal, glycemic, and lipid profiles were accessed, as well as systemic and ovarian oxidative status; BW, cyclicity, ovarian histomorphology, ovarian volume, testosterone and progesterone levels, as well as LDL, triglycerides, and total cholesterol levels were aggravated after PCOS-induction and improved after MET, SEA, and MET + SEA treatment. MET + SEA had the greatest impact on glycoregulation. Alterations in OS parameters (TBARS, O_2_^−^, H_2_O_2_, catalase, superoxide dismutase, and reduced glutathione) could be responsible for observed differences; (4) Conclusions: Our findings confirmed that SAE alone or along with MET was capable of ameliorating reproductive and metabolic disturbances in the PCOS rat model, with the restoration of OS parameters. SAE alone did not alter the protective effects of MET in PCOS.

## 1. Introduction

Polycystic ovary syndrome (PCOS) represents one of the most common endocrinopathies among childbearing-age women; 5–20% of this population have PCOS [1]. Although PCOS exerts very serious reproductive, metabolic, cardiovascular, and psychological properties, this complex syndrome frequently remains undiagnosed. The reason for this lapse could be the heterogeneity of presented symptoms, inconsistency of diagnostic criteria applied, or even misunderstanding of its severity from the patients and physicians.

From the time when it was first recognized, in early 1935, PCOS underwent various vestures. These modifications made PCOS phenotypes; from obese women with polycystic ovaries to lean ones with no polycystic ovarian morphology [2,3]. Moreover, hyperandrogenism (clinical or biochemical) is another characteristic that is not even obligatory for PCOS diagnosis precisely because there are different phenotypes expressed in this complex syndrome with a plethora of characters.

The etiopathogenesis of PCOS is extremely complicated, while its management remains controversial. Insulin resistance (IR) and compensatory hyperinsulinemia were estimated to be present in 65–70% of PCOS women and appear to underlie many of its endocrine features [4]. In the last decades, science recognized a rush of lean PCOS subjects with hyperlipidemia and hyperinsulinemia; obese or even lean women could experience IR, which, in turn, influences testosterone levels and worsens hyperandrogenemia in PCOS [5]. The explanation for this vicious circle lies in the fact that compensatory hyperinsulinemia could increase the sensitivity of granulosa cells to luteinizing hormone (LH) and further increase androgen release by theca cells while decreasing sex hormone-binding globulin production by the liver. Moreover, higher insulin levels shift the follicle-stimulating hormone (FSH) to LH, preventing the selection of a dominant follicle [6]. Nowadays, there is no specific targeting therapy to combat PCOS efficiently. However, numerous protocols have been used in PCOS treatment, beginning with lifestyle modification, including diet and exercise [7], supplementation with insulin-sensitizing agents (such as myo and D-chiro-inositol [8], metformin (MET) therapy, and oral contraceptive pills administration [9]. In addition, various studies investigated the role of alternative medicine in helping alleviate PCOS symptoms [10]. Nevertheless, a curative protocol has not been proposed yet, and all applied interventions remained mostly symptomatic.

The complexity of PCOS pathogenesis and the heterogeneity of its symptoms make it difficult to target the underlying mechanism responsible for the development of such a condition. Apart from IR, oxidative stress (OS) along with low-grade inflammation might further unpin PCOS pathophysiological mechanisms [11]. These assertions could justify the existence of numerous studies (clinical and preclinical) investigating the role of different antioxidative and anti-inflammatory agents in PCOS management. Although animal PCOS models do not entirely mimic the real PCOS settings in women, these models could help in lightening the specific pathophysiological alterations in local and systemic levels regarding PCOS [12]. Such investigations leave evidence and give rise to further clinical investigations in order to improve therapeutic strategies to combat PCOS symptoms.

*Aronia melanocarpa*, usually alluded to as black chokeberry, belongs to the *Rosaceae* family, and was presented to Eastern Europe from North America. The antioxidant activity of black chokeberry was recognized in many studies [13]. Containing the highest level of polyphenols among fruit [14], it represents an attractive candidate for coping with various OS-related pathologies [15,16]. Our previous investigations confirmed the ameliorative effect of *Aronia melanocarpa* (administered as a standardized extract of *Aronia melanocarpa*—SEA) regarding metabolic syndrome in rats [17]. Furthermore, our previous results even confirmed the protective role of SEA in hemodialysis patients with anemia [18]. Interestingly, SEA improved clinical and biochemical parameters in patients with confirmed metabolic syndrome even to a greater extent in women compared to men [19], probably involving the role of sex hormones on lipid metabolism. These data led us to an idea to explore the potential benefits of SEA administration in an androgen-induced PCOS rat model previously established in our laboratory [20,21]. Moreover, we aimed to investigate the synergistic effects of SEA and MET therapy in alleviating reproductive and metabolic abnormalities of PCOS.

## 2. Materials and Methods

### 2.1. Ethics Statement

This investigation was conducted at the Faculty of Medical Sciences, University of Kragujevac (Kragujevac, Serbia), and the Faculty’s Ethical Committee for experimental animal well-being approved ethical permission (number 01-17057). All procedures were carried out in accordance with the European Council Directive (No. 86/609/EEC), principles of the Good Laboratory Practice, and ARRIVE guidelines. Every effort has been made to reduce the number of animals used and their suffering.

### 2.2. Animals

Adult Wistar albino female rats (*n* = 42, body weight (BW) 150–170 g) were obtained from the Military Medical Academy (Belgrade, Serbia). All animals were housed under controlled conditions (temperature of 23 ± 1 °C, 12:12 h light/dark cycle—lights on 08:00 h), with access to food and water ad libitum. After acclimatization, all animals underwent estrus cycle examination, and only those with regular (4–5 days) cycling were enrolled in the experimental protocol.

### 2.3. Experimental Protocol

#### 2.3.1. Assessment of Estrous Cycle

The estrus cycle was assessed by cytological examination of vaginal smears. In the morning (between 9:00–10:00 h (h)), vaginal lavage was performed by using a dropper filled with a small volume of saline, placed on a glass slide, stained with hematoxylin, and analyzed by a light microscope. Estrus cycle phases were identified by predomination of specific cells, as follows: proestrus—round, nucleated cells; estrus—cornified squamous cells; metaestrus—cornified squamous cells; and leucocytes and diestrus—nucleated epithelial cells and predominance of leucocytes [22]. An assessment of the estrous cycle was performed over 12 consecutive days.

#### 2.3.2. PCOS Induction

All regularly cycling rats were randomly divided into two groups—control (C, *n* = 9) and PCOS group (PCOS, *n* = 33). PCOS was induced by daily subcutaneous injections of dehydroepiandrosterone (DHEA, Millipore, Darmstadt, Germany; 60 mg/kg of BW) dissolved in 0.2 mL of sesame oil (Sigma-Aldrich, St. Louis, MO, USA) for 35 days [23]. During the experimental protocol, BW was measured daily to calculate the drug doses. During the same period, animals from the C group received 0.2 mL of sesame oil. To minimize the effect of manipulation on animal welfare, the injections were given every day at the same interval (11:00–11:30 h).

In the last 12 days, the animals underwent estrus cycle examination as described above. After completion of the experimental protocol, three randomly chosen animals from both C and PCOS groups were sacrificed to confirm successful PCOS modeling. PCOS was confirmed as estrus cycle cessation, and the presence of ovarian cysts (OC) with the absence of corpora lutea (CL) was assessed by histological analysis of ovarian tissue sections.

#### 2.3.3. Grouping and Treatments

**Figure 1:**
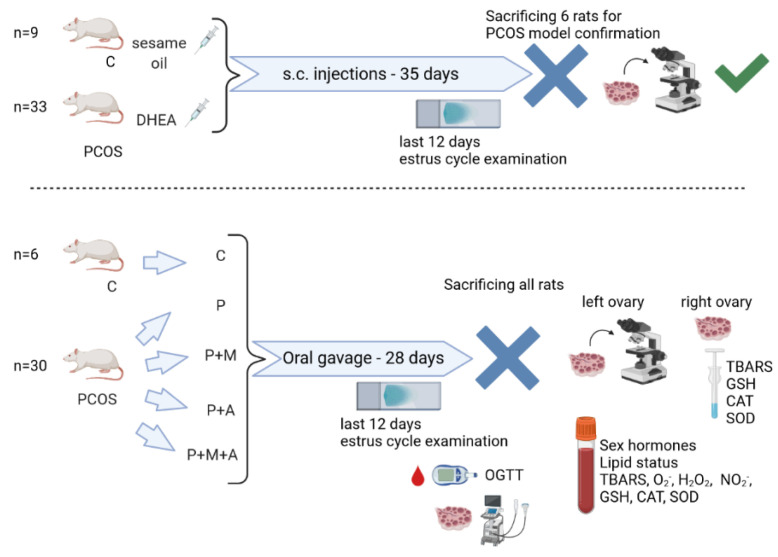
Schematic diagram of the rat study protocol, grouping, treatments, and measurements performed.

The remaining animals, 6 from C and 30 from the PCOS group, were enrolled in further investigation. The PCOS group was divided into the following subgroups: P (*n* = 6, animals treated by gavage with distilled water), P + M (*n* = 6, animals treated by gavage with MET), P + A (*n* = 6, animals treated by gavage with SEA), and P + M + A (*n* = 6, animals treated by gavage with MET and SEA). MET (Sigma, Aldrich) was dissolved in distilled water and administered at a dose of 500 mg/kg of BW [24] daily, while SEA (Pharmanova, Belgrade, Serbia) was administered at a dose of 0.45 mL/kg of BW [17], daily. All study protocols lasted for 28 days. During the last 12 days, the estrus cycle examination was performed, as described above. The grouping and treatments are presented in Figure 1.

##### *Aronia melanocarpa* Standardized Extract

SEA represents a product of the pharmaceutical company Pharmanova (Belgrade, Serbia). EU-Chem (Belgrade, Serbia) performed the procedure of SEA extraction. Chemical analysis of SAE for the identification and quantitation of flavonoids and anthocyanins was performed using the high-performance liquid-chromatography diode array detector (HPLC-DAD) based on C-18 reversed-phase column separation. The following individual compounds were found in SAE: 2.68 cyanidin 3-galactoside (80.40 mg/mL), 0.16 cyanidin 3-glucoside (4.92 mg/mL), 0.66 cyanidin 3-arabinoside (19.71 mg/mL), 0.14 cyanidin 3-xyloside (4.26 mg/mL), 0.12 rutin (3.55 mg/mL), 0.27 hyperoside (8.12 mg/mL), and 0.15 isoquercetin (4.36 mg/mL). The total amount of polyphenols was 410 mg/30 mL of SAE with a total energy value of 502.47 kJ (118.23 kcal)/100 mL [19].

### 2.4. Oral Glucose Tolerance Test (OGTT)

Forty-eight hours before sacrifice, all animals were exposed to overnight fasting for 12 h, and afterward, glucose (2 g/kg) was administered by gavage. The blood samples were obtained from the tail vein before and at 30, 60, 120, and 180 min after glucose administration. The glucose levels were determined using a glucometer (Accu-Chek, Roche Diagnostics, Indianapolis, IN, USA) with corresponding strips.

### 2.5. Ultrasound Examination of Ovaries

At the end of the protocol, an ultrasound examination of the left ovary was performed using Hewlett Packard Sonos 5500 (Andover, MA, USA) equipped with a 15.0 MHz phased-array linear transducer. First, all rats were anesthetized using a ketamine (50 mg/kg) and xylazine (10 mg/kg) mixture. Using a transabdominal view in 2-dimensional B-mode, the ovaries were localized topographically beside kidneys and towards echogenic characteristics. Three dimensions of the ovary were measured, and ovary volume (mm^3^) was calculated by the formula for a prolate ellipsoid, where the volume is the product of π/6 and the longitudinal (LOD), transverse (TOD), and anteroposterior (APOD) ovarian diameters [volume = π/6 (LOD × TOD × APOD)] [25].

### 2.6. Sacrificing the Animals and Sample Collection

After completion of the study protocol, all animals were anesthetized by intraperitoneal application of a ketamine (10 mg/kg) and xylazine (5 mg/kg) mixture and sacrificed by decapitation. The trunk blood samples were collected, and the obtained serum, plasma, and erythrocyte lysate samples were stored at −20 °C for further analysis. The ovaries were isolated and prepared for histological (left ovary) and biochemical (right ovary) analysis. All animals were sacrificed at the same estrus cycle phase (diestrus) in order to eliminate the impact of the cycle phase on the analyzed parameters.

#### Ovarian Tissue Homogenization

The right ovaries were resected from connective tissue, measured, and rinsed in ice-cold phosphate-buffered saline (pH 7.4). A 1/10 weight/volume ratio was set for homogenization of the ovary, and the homogenate was centrifuged at 1200× *g* at 4 °C for 20 min. The supernatants were stored at −70 °C until the analyses were performed [26].

### 2.7. Biochemical Analysis

#### 2.7.1. Hormonal Assays

Serum samples were used for the assessment of the following sex hormone levels: total testosterone (T), progesterone (P4), and estradiol (E2). T, P4, and E2 levels were determined by an Elecsys 2010 analyzer using the method of the electrochemiluminescence immunoassay (ECLIA). Standard commercial kits (Elecsys Testosterone II, Progesterone II, and Estradiol III Roche Diagnostics, Mannheim, Germany) were used, and the T and P4 levels were expressed in ng/mL, while E2 was expressed in pg/mL. The sensitivities of the assays for T, P4, and E2 were 0.025 ng/mL, 0.03 ng/mL, and 5 pg/mL, respectively. Inter and intra-assay coefficients of variance for T, P4, and E2 were 3.8%, 3%, and 2.2%, and 5%, 5%, and 3.9%, respectively.

#### 2.7.2. Lipid Profile

The serum levels of the total cholesterol (TC), triglycerides (TG), and high and low-density lipoprotein (HDL and LDL) were determined spectrophotometrically on a programmed biochemical analyzer (Dimension Xpand, Siemens, IL, USA) using commercial kits (Siemens Healthcare Diagnostics, Frimley, Camberley, Surrey, UK) [27].

#### 2.7.3. OS Parameters

In the plasma samples, the following parameters were determined: index of lipid peroxidation (expressed as thiobarbituric acid reactive substances—TBARS), level of nitrites (NO_2_^−^), hydrogen peroxide (H_2_O_2_), and superoxide anion radical (O_2_^−^). The activity of enzymatic (superoxide dismutase (SOD) and catalase (CAT)), as well as non-enzymatic (GSH) antioxidant systems, were determined from the lysed erythrocyte suspension. In ovarian tissue homogenates, TBARS, SOD, CAT, and GSH were measured.

##### TBARS Determination

The procedure was performed by mixing 0.8 mL of the sample with 0.4 mL trichloroacetic acid. After 15 min on ice and centrifugation at 6000 rpm, the supernatant was collected. Incubation of 1% thiobarbituric acid in 0.05 NaOH occurred with the supernatant at 100 °C for 15 min. The measurement was performed at 530 nm of wavelength. A distilled water solution served as a blank probe [28].

##### Determination of NO_2_^−^

The NO2- level was determined as an index of NO production with Griess reagent [29]. A total of 0.1 mL 3 N perchloride acid, 0.4 mL 20 mM ethylenediaminetetraacetic acid, and 0.2 mL of the sample were kept on ice for 15 min and then centrifuged for 15 min at 6000 rpm. After pouring off the supernatant, 220 µL K_2_CO_3_ was added. NO_2_^-^ was measured at a wavelength of 550 nm, and distilled water served as a blank probe.

##### Determination of H_2_O_2_

The determination of hydrogen peroxide (H_2_O_2_) was based on the oxidation of phenol red by hydrogen peroxide in a reaction catalyzed by horseradish peroxidase (HRPO). A total of 200 µL of plasma sample was precipitated with 800 µL of freshly prepared phenol red solution, followed by the addition of 10 µL of (1:20) HRPO. The level of H_2_O_2_ was measured at a wavelength of 610 nm, and distilled water served as a blank probe [30].

##### Determination of O_2_^−^

The concentration of the superoxide anion radical (O_2_^−^) was measured after the reaction of nitro blue tetrazolium in the TRIS buffer with the plasma samples. The determination was performed at a wavelength of 530 nm. A distilled water solution served as a blank probe [31].

##### Determination of CAT

A total of 50 µL of CAT buffer, 100 µL of the sample, and 1 mL of 10 mM H_2_O_2_ were used for CAT determination. The detection was performed at a wavelength of 360 nm. Distilled water served as a blank probe, and the amount of CAT was expressed as U/g tissue or U/g of hemoglobin × 10^3^ [32,33].

##### Determination of SOD

SOD activity was detected by the epinephrine method by Misra and Fridovich [34]. A total of 100 µL of sample and 1 mL of carbonate buffer were mixed, and then 100 µL of epinephrine was added. Detection was performed at a wavelength of 470 nm, and the amount of SOD was expressed as U/g tissue or U/g of hemoglobin × 10^3^.

##### Determination of GSH

The level of reduced glutathione (GSH) was based on GSH oxidation via 5,5-dithiobis-6,2-nitrobenzoic acid. GSH extract was obtained by combining 0.1 mL of 0.1% EDTA, 400 µL plasma, and 750 µL precipitation solution (containing 1.67 g metaphosphoric acid, 0.2 g EDTA, 30 g NaCl, and filled with distilled water until 100 mL). After mixing in the vortex machine and extraction on ice for 15 min, it was centrifuged at 4000 rpm (for 10 min). Measuring was performed at a wavelength of 420 nm, while distilled water served as a blank probe [35].

#### 2.7.4. Determination of Total Protein Content

The total protein content of tissue samples was quantified by the modified Lowry’s method [36].

### 2.8. Ovarian Histology

After sacrificing the animals, the left ovaries were excised, cleaned, measured, fixed in 10% neutral buffered formalin solution, and processed for light microscopic analysis, dehydrated in ascending grades of alcohol, cleared in xylene, and embedded in paraffin. The tissue sections (4 µm thin) were stained with hematoxylin and eosin. Central ovarian sections were used to evaluate the ovarian histomorphology; the number of cystic follicles and number of CL were determined using Olympus BX-51, Olympus Europa GmbH, Hamburg, Germany [20].

### 2.9. Statistical Analysis

Values were presented as the mean ± standard error. Before statistical analysis, all data were checked for normality and, depending on distribution; the data were evaluated using ANOVA of Kruskal–Wallis test, with appropriate post-hoc analysis. These analyses were carried out using SPSS statistical program version 22.0. A *p*-value below 0.05 was considered to be statistically significant, and a *p*-value below 0.01 was considered to be highly statistically significant.

## 3. Results

### 3.1. Effect of MET, SEA, and Their Combination on PCOS-Related Anthropometric Indices, Estrus Cycle, and Ovarian Histology

**Figure 2:**
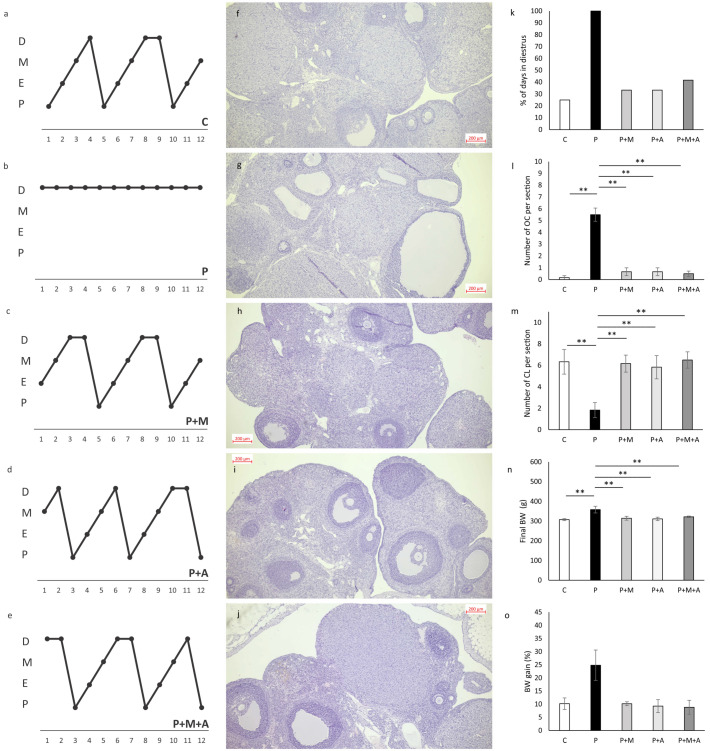
Characteristics of the PCOS rat model. ((**a**) C group, (**b**) P group, (**c**) P + M group, (**d**) P + A group, (**e**) P + M + A group) Estrus cycle, P—proestrus, E—estrus, M—metaestrus, and D—diestrus; ((**f**) C group, (**g**) P-group, (**h**) P + M group, (**i**) P + A group, (**j**) P + M + A group) Histomorphological evaluation of the central ovarian section (magnification 5×, scale bar = 200 µm); Percentage of days in diestrus (**k**); Number of ovarian cysts (OC) per section (**l**); Number of corpora lutea (CL) per section (**m**); Final body weight (BW) (**n**); Percentage of BW gain (**o**). Data are presented as mean ± SEM. ** Statistical significance at the level of *p* < 0.01 between groups (6 rats per group).

As shown in Figure 2, only the P group exerted a persistent diestrus phase (Figure 2b), while all remaining groups showed normal ovarian cyclicity (Figure 2a,c–e). The percent of days in the diestrus phase was significantly higher in the P group compared to all other investigated groups (Figure 2k). After all three types of applied treatment, the cycle was restored to regular after 4–5 days of duration.

Ovarian histological sections (Figure 2f) depicted normal ovarian morphology, follicles in the different stadiums of folliculogenesis, and many of CL in the C group. On the other hand, the P group showed the presence of OC with very thin granulosa cells layers, decreased number of CL, and enlargement of ovarian stroma (Figure 2g). As shown in Figure 2h–j, all treated groups (P + M, P + A, and P + M + A, respectively) are characterized by restored ovarian morphology. These groups showed a markedly decreased number of follicular cysts (Figure 2l), and an increased number of CL (Figure 2m) compared to the P group. Ovaries from groups supplemented with MET, SEA, or their combination were visualized as structures with lesser atretic follicles and ovarian stromal tissue and an increased number of healthy antral and tertiary follicles.

Final BW was the highest in the P group and significantly differed (*p* < 0.01) from all other groups (Figure 2n). All three kinds of treatment decreased final BW when compared to the saline-treated P group. The percentage of BW gain was largest in the P group compared to all other investigated groups (as shown in Figure 2o).

### 3.2. Effect of MET, SEA, and Their Combination on PCOS-Related Ovarian Dimensions Assessed by Ultrasonographical Analysis

**Figure 3:**
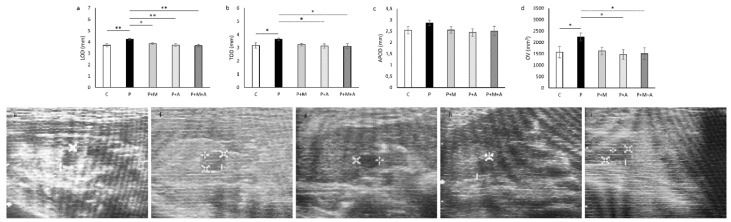
Ultrasonographic evaluation of ovarian measurements in rats. (**a**) Longinudinal ovarian diameter (LOD); (**b**) Transversal ovarian diameter (TOD); (**c**) Anteroposterior ovarian diameter (APOD); (**d**) Ovarian volume (OV); Representative ultrasonographical images of left rat ovaries ((**e**) C group, (**f**) P group, (**g**) P + M group, (**h**) P + A group, (**i**) P + M + A group). Data are presented as mean ± SEM. * Statistical significance at the level of *p* < 0.05 between groups, ** Statistical significance at the level of *p* < 0.01 between groups (6 rats per group).

Ultrasonographycally measured ovarian dimensions were different between investigated groups: LOD and TOD were elevated (*p* < 0.05) in the P group when compared to the C group (Figure 3a,b). LOD was significantly decreased in the P + M (*p* < 0.01), P + A (*p* < 0.01), and P + M + A (*p* < 0.01) compared to the P group, while TOD was decreased only in the Aronia-treated groups (*p* < 0.05). APOD was not altered between investigated groups (Figure 3c). Ovary volume was increased (*p* < 0.05) in the P compared to the C and Aronia-supplemented groups (Figure 3d). In all investigated groups, ovaries are shown as oval, clearly demarcated isoechoic formations according to ultrasound characteristics. In the P group (Figure 3f), hypoechoic circular formations could be observed (which could correspond to cysts), as well as the foci of atretic follicles and hyperthecosis. However, C, P + M, P + A, and P + A groups (Figure 3e,g–i, respectively) appeared as normal ovarian structures without fluid-filled formations and hyperthecosis.

### 3.3. Effect of MET, SEA, and Their Combination on PCOS-Related Changes in Serum Sex-Hormone Levels

As presented in Table 1, T levels were increased in the P group compared to the C and all three treated groups (*p* < 0.01). E2 levels were similar between C and P groups, but SEA and MET treatment, as well as their combination, decreased (*p* < 0.05) levels of this hormone compared to the P group. P4 levels were decreased in the P group compared to control values (*p* < 0.05), but treatment with SAE, MET, and their combination elevated its levels (*p* < 0.01, *p* < 0.05, and *p*< 0.01, respectively), toward control values.

### 3.4. Effect of MET, SEA, and Their Combination on PCOS-Related Alterations in Glycoregulation

**Figure 4:**
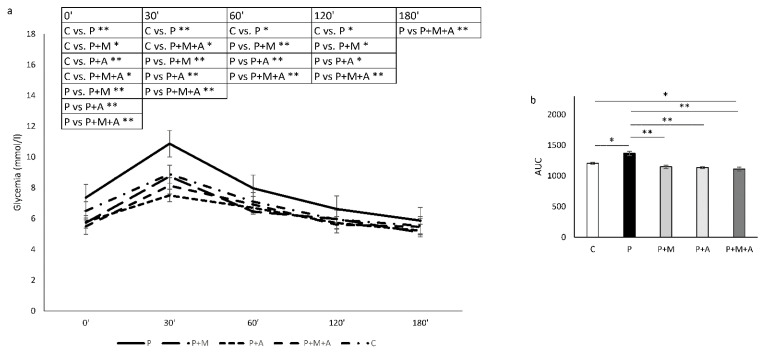
Results of oral glucose tolerance test in rats. (**a**) Glucose concentration in plasma at 0, 30, 60, 120, and 180 min. (**b**) Area under the curve (AUC). Data are presented as mean ± SEM. * Statistical significance at the level of *p* < 0.05 between groups, ** Statistical significance at the level of *p* < 0.01 between groups (6 rats per group).

A significant increase in basal glucose levels was evident in the P group compared to all investigated groups (*p* < 0.01), as shown in Figure 4a. However, basal glucose levels in P + M, P + A, and P + M + A groups were decreased even compared to control values, but none of the rats from the mentioned groups was in a hypoglycemic state. After glucose overload, significantly increased glycemic values in the P group persisted in 30′, 60′, and 120′ compared to all investigated groups, while in 180′ there was only a difference between the P and P + M + A groups (*p* < 0.01).

The area under the curve (AUC) was increased in the P compared to C (*p* < 0.05) and other groups (*p* < 0.01), as shown in Figure 4b. Moreover, the difference was also registered between C and P + M + A groups (*p* < 0.05) regarding this parameter.

### 3.5. Effect of MET, SEA, and Their Combination on PCOS-Related Changes in Serum Lipid Profile

As shown in Table 2, total cholesterol values were increased in the P compared to C (*p* < 0.01), P + M (*p* < 0.01), P + A (*p* < 0.05), and P + M + A groups (*p* < 0.01). Serum triglyceride and LDL cholesterol levels were elevated in the P compared to all investigated groups (*p* < 0.01). HDL levels were unaltered between investigated groups, with a slight tendency in P + A and P + M groups toward elevated HDL values.

### 3.6. Effect of MET, SEA, and Their Combination on PCOS-Related Alteration of Systemic Oxidative Status

**Figure 5:**
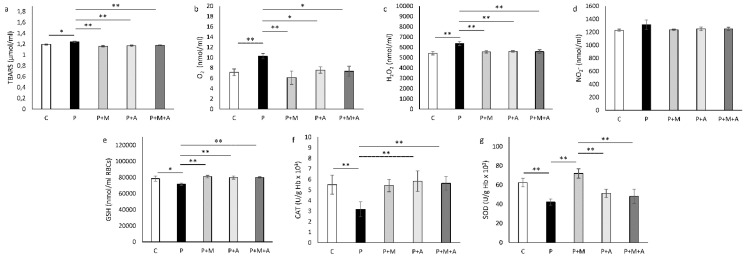
Oxidative stress parameters in the blood of rats. (**a**) Index of lipid peroxidation (TBARS); (**b**) Levels of superoxide anion radical (O_2_^−^); (**c**) Levels of hydrogen peroxide (H_2_O_2_); (**d**) Levels of nitrites (NO_2_^−^); (**e**) Level of reduced glutathione (GSH); (**f**) Activity of catalase (CAT); (**g**) Activity of superoxide dismutase (SOD). Data are presented as mean ± SEM. * Statistical significance at the level of *p* < 0.05 between groups, ** Statistical significance at the level of *p* < 0.01 between groups (6 rats per group).

Pro-oxidative parameters, TBARS, O_2_^−^, and H_2_O_2_, were significantly increased in the P compared to the C group (*p* < 0.01, *p* < 0.01, and *p* < 0.05, respectively), as shown in Figure 5a–c. Treatment with MET significantly decreased all these parameters compared to the P group (*p* < 0.01). Aronia-supplemented groups showed decreased O_2_^−^ levels (*p* < 0.05) and also H_2_O_2_ and TBARS (*p* < 0.01). Combined SAE and MET treatment led to a decrease of TBARS, O_2_^−^, and H_2_O_2_ levels compared to P group (*p* < 0.05, *p* < 0.05, and *p* < 0.05, respectively). Levels of nitrites were unaltered (Figure 5d).

GSH values were decreased in the P group (*p* < 0.05) in comparison to the C group, while all treated groups showed elevation of GSH levels compared to the P group (*p* < 0.01), as shown in Figure 5e.

CAT levels were decreased in the P group (*p* < 0.01) in comparison to the C group. Although the MET-treated group did not show any change compared to the P group, both Aronia-treated groups showed elevation of CAT activity (*p* < 0.01) compared to the P group (Figure 5f).

The antioxidant capacity of SOD was decreased in the P group compared to the C group (*p* < 0.01), while treatment with MET increased SOD activity (*p* < 0.01) toward control values (Figure 5g). Aronia-treated groups (P + A and P + M + A) did not differ from the P group regarding SOD activity, but showed lower SOD levels in comparison to the P + M group (*p* < 0.01).

### 3.7. Effect of MET, SEA, and Their Combination on PCOS-Related Alteration of Ovarian Oxidative Status

**Figure 6:**
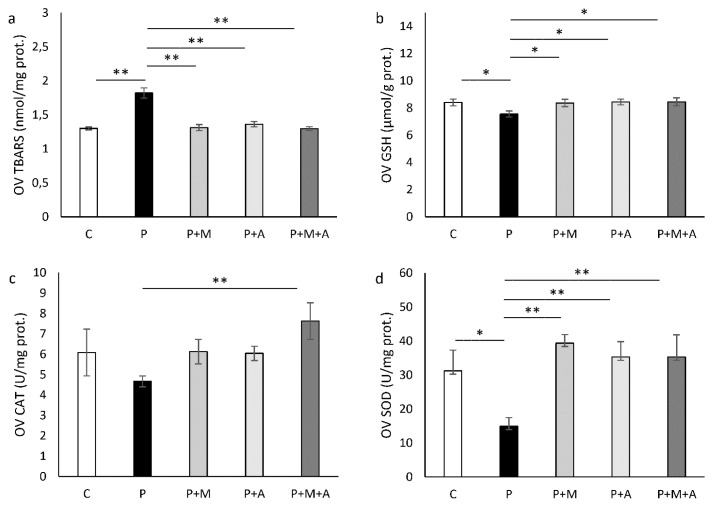
Oxidative stress parameters in ovarian tissue. (**a**) Index of lipid peroxidation (TBARS); (**b**) Level of reduced glutathione (GSH); (**c**) Activity of catalase (CAT); (**d**) Activity of superoxide dismutase (SOD). Data are presented as mean ± SEM. * Statistical significance at the level of *p* < 0.05 between groups, ** Statistical significance at the level of *p* < 0.01 between groups (6 rats per group).

Ovarian TBARS levels were increased in the P group (*p* < 0.01) in comparison to the C group. However, treatment with SEA, MET, or their combination decreased these parameter values (*p* < 0.01) compared to the P group (Figure 6a).

As shown in Figure 6b, GSH values were decreased in the P group compared to all other investigated groups (*p* < 0.05).

CAT activity was increased only in the P + M + A group (*p* < 0.01) compared to the P group (Figure 6c).

Ovarian SOD activity was decreased in the P group (*p* < 0.05) in comparison to the C group, and all treated groups showed elevation of SOD compared to the P group (*p* < 0.01), as shown in Figure 6d.

## 4. Discussion

The present study investigated the effects of SAE, MET, or their combination on metabolic and reproductive characteristics of the DHEA-induced PCOS rat model. Our results strongly confirmed the role of SAE alone or in combination with MET in ameliorating PCOS-related abnormalities. The BW, estrus cycle, ovarian morphology, glycemic, lipid, and hormonal status were altered after PCOS induction, while SAE, MET, as well as their combination improved these PCOS-related alterations. OS parameters were disturbed in rats with PCOS, while all three types of treatment improved systemic and ovarian oxidative status and combated the worsening of specific parameters involved in the molecular basis of PCOS-related reproductive and metabolic disturbances. Besides a lot of evidence suggesting beneficial effects of *Aronia melanocarpa* in OS-related disorders [16], to the best of our knowledge, in the present literature, there is no similar investigation conducted on the PCOS rat model. Our study showed the beneficial effects of *Aronia melanocarpa* extract administration in the PCOS rat model and its associated metabolic characteristics for the first time.

We successfully developed the PCOS rat model by following the method of Kim and coworkers [23], which exert very similar features to those seen in PCOS patients. All rats from the P group remained acyclic, in the persistent diestrus phase, during the last 12 days of the PCOS induction protocol. That feature is common for rat PCOS models and represents a sign of ceased/impaired cyclicity and absent/less frequent ovulation [37,38]. However, after administration of MET, as a frequently used medicine for cycle regulation and ovulation induction in women, the rats recovered their cycle to regular (4–5 days of duration), as expected [39]. Interestingly, SEA alone, or in combination with MET, also improved cyclicity in all treated groups implying that these agents had a similar mechanism of action in restoring normal cycle duration in the PCOS-induced model. However, there are no studies to compare these results regarding *Aronia melanocarpa*, neither in animal models nor in women. Therefore, we could only suggest further experimental studies with different PCOS modeling, as well as clinical investigations, to verify the effects of *Aronia melanocarpa* in oligo and an-ovulatory PCOS patients.

Aside from the cycle, ovarian histological analysis revealed successful PCOS induction observed as multiple follicular cysts developed in the P group with a lowered number of CL, which suggests a decrease in ovulation number. Represented lower number of old CL in the P group, few developing follicles with thicker granulosa layer expressing periodical detachments of layers and cumulus mass, and enlarged stroma could be interpreted as ovarian dysfunction, as seen in PCOS [40]. Furthermore, an improvement in ovarian morphology was found after SEA and MET treatment, alone or in combination, in the sense of a decreased number of cysts, showing follicles at different stages of growth, and a larger number of CL compared to the P group, which definitely pointed to beneficial effects of both applied protocols. The presented photomicrographs showed reversing effects of applied treatment in cystic ovarian appearance, which represents only one aspect of PCOS, but is accompanied by other results assembled in a mosaic of its complexity.

Although not all preclinical studies confirmed BW gain after PCOS induction, our results showed that the post-pubertal DHEA-induced model mimics the obese PCOS phenotype, as others confirmed [41]. On the other hand, pre-pubertal DHEA-induced PCOS models frequently showed an unaltered BW compared to non-PCOS groups [42], which is one more advantage of the applied post-pubertal DHEA protocol in our study. As shown in Figure 2n, a final BW was the largest in the P group, while all three treatments decreased BW gain toward control levels. This effect of MET on weight loss was shown in both PCOS women [43] and animal studies [44]. Furthermore, there was a confirmed effect of SAE administration on BW loss in male rats with metabolic syndrome [17], as well as in male and female patients with metabolic syndrome and diabetes mellitus [19]. In the mentioned study, 2 weeks of SAE treatment significantly reduced BW in male diabetic patients, while after 4 weeks of SEA administration, BW was significantly reduced in both female and male patients. Our study showed that the 4-week protocol of SAE successfully reduced BW to control values in PCOS treated groups. There are controversies in the literature regarding BW loss and polyphenols in diets, probably due to different protocols applied and confounding factors, such as clinical or subclinical comorbidities in humans [45,46]. However, animal studies confirmed positive effects on weight loss after the consumption of polyphenol-rich chokeberry extract [47] by modulating multiple pathways associated with insulin signaling, adipogenesis, and inflammation, which certainly could be beneficial in PCOS management.

Earlier studies showed that up to 22% of women have ultrasonographically confirmed polycystic ovarian morphology, but only 5–10% developed the syndrome, with all or most of the accompanying features [48]. Ultrasonographical evaluation of rats’ ovaries depicted increased OV in the P group (by 42.34%). It is known that broadened ovaries with numerous little follicles incidentally found around expanded ovarian stroma with expanded stromal echogenicity are the sonographic highlights of polycystic ovaries in women [49]. However, in our previous study, chronic testosterone enanthate treatment led to a marked decrease in OV [21], as a more potent androgen from DHEA and with different metabolic pathways. Similar results were obtained by—probably the most precise tool for organ volume measurement—the morphometric Cavaliery method, used by Kalhori and coworkers [50]. Taken together, the DHEA-induced PCOS rat model used in our study exhibited more similar polycystic ovarian morphology with enlarged ovaries to those seen in PCOS women, suggesting this model is superior to others regarding mimicking human ovarian morphology during PCOS. A decrease in OV and TOD was superior when SAE was applied alone or in combination with MET, contrary to MET alone, which did not show any effect on decreasing these parameters. Moreover, although significant, a decrease in LOD by MET was only 8.94%, compared to the more pronounced effect of SAE and SAE combined with MET (12.24% and 13.18%, respectively). These alterations supported SAE as superior to MET alone in reversing PCOS-related ovarian ultrasonographical parameters.

A typical hyperandrogenemic hormonal milieu has been observed after PCOS induction in our study. An elevation in T levels, as a prominent PCOS characteristic, was also recognized in a similar investigation [51], where a clear effect of MET treatment in decreasing T levels was shown. Interestingly, both agents, SAE and MET, alone or in combination, significantly decreased levels of T in our experiments. As mentioned previously, there are no data regarding the effects of *Aronia melanocarpa* on hyperandrogenemia in females. However, there is one experimental study showing lowering effects of *Aronia melanocarpa* extract on expression levels of dihydrotestosterone levels in prostate tissue and a tendency for a decreased serum dihydrotestosterone and 5-alpha reductase level in serum, attributing the effects to high anthocyanin and phenolic content in the applied extract [52]. However, no alteration in the E2 level was found after PCOS modeling, although MET, SAE, and their combination significantly decreased E2 levels compared to the P group. It was known that anthocyanins, such as cyanidin-3-glucoside from blackberry, could exert a phytoestrogenic effect [53], binding with estrogenic receptors alpha and beta [54]. On the other hand, there are studies showing that potent soy isoflavones, such as genistein and daidzein, also exert phytoestrogenic activity but without altering the E2 serum levels in the PCOS rat model [55]. A possible explanation may be proposed if SAE, as well as MET [56], could block or decrease aromatase activity and thus prevent androgenic as well as estrogenic PCOS properties. During PCOS pathophysiology, the hypothalamic-pituitary-gonadal axis gets disturbed, leading to altered hormonal secretion such as increased levels of T and LH [57]. The elevated T level stimulates the secretion of LH in the thecal follicles through the stimulation of the PI3K/Akt pathway [58]. The PI3K pathway is a moderator of LH-dependent Akt phosphorylation in follicles, up-regulates the ovarian CYP17A1 gene expressions, and further elevates the activity of the 17-α hydroxylase enzyme. Because 17α-hydroxylase is a core enzyme that catalyzes the steroidogenic conversion of progesterone into androgens, it increases the level of androgens. This circuit could also be seen regarding the T/P4 relationship and their inverted/mirrored levels regarding MET or SAE treatment after PCOS induction in our study. The CYP17a1 and CYP11a1 genes encode 17-α hydroxylase and 17,20-lyase, respectively, two key enzymes involved in the synthesis and metabolism of androgens. In androgenized rats, there was MET-induced CYP17 expression improving ovarian follicular dynamics [59], which could be particularly presumed to take place in our study. One of the limitations of our study was the technical inability to measure the activity of mentioned enzymes involved in steroidogenesis, and it is a plan for future investigations regarding the administration of SAE in PCOS rats in order to obtain a deeper insight into the molecular basis of observed hormonal alterations.

Regarding glycemic control, SAE exerted as much anti/hypoglycaemic effects as expected. Even as it is not yet investigated in PCOS settings, as in many studies, including metabolic syndrome [16], we could assume beneficial effects after SAE treatment in our study. Moreover, the PCOS equivalent is recognized in males also [60,61], especially in the case of men with PCOS-positive family history and hyperandrogenism signs accompanied by the metabolic alterations and hormonal PCOS patterns. As mentioned above, in our previous study, male rats supplemented with SAE at the same dose used in this study had an improved glucose tolerance in the state of metabolic syndrome [17]. Moreover, SEA did not affect glycemia in healthy rats, but it did lower insulin levels. In the present study, rats from the P group showed increased baseline glycemia compared to the C group, as expected [40], but to all other groups also. Interestingly, SAE and MET treatments, alone and when combined, lowered the baseline glycemia even when compared to untreated rats, but none of them exhibited hypoglycemic status. After 30 min of glucose overload, the P group continued to be increased compared to other groups, while only SAE and MET combination remained below control values at this time point measurement. This manifestation suggests synergistic effects of SAE and MET in combating the overload of glucose in the early stage, improving metabolism while disappearing after 60, 120, and 180 min, probably preventing further hyperglycemic episodes. Most interestingly, after 180 min of glucose administration, only SAE in combination with MET showed significantly lower glucose levels compared to the P group, favoring the joint effects of the two applied agents. Moreover, AUC analysis expressed higher values in the P group compared to all other groups, while among treated groups, only SAE combined with MET showed decreased AUC compared to the C group. This result should certainly be considered when discussing improved glycoregulation, particularly as prevention of further metabolic complications concomitantly expressed with PCOS in later life. In spite of the fact that MET exerts a modest impact on PCOS and PCOS-related obesity, it is still the pillar of restorative treatment. Therefore, it is worth proposing novel strategies in order to improve as many aspects as well regarding the plethora of PCOS symptoms. Moreover, Aronia is shown to act by the inhibition of α-glucosidase and dipeptidyl peptidase IV [62], which is shown to be superior when combined with MET in comparison to MET alone in the management of PCOS [63]. Taken together, these claims should be directed to further clinical examinations combining SAE and MET in PCOS patients, particularly those with obese phenotypes. MET was shown to improve body composition and insulin levels in women with PCOS who are not obese but has no significant effect on body mass index, fasting glucose, or lipid levels [64], so its combination with SAE could be considered valuable. PCOS is a multisystem disorder with tissue-selective insulin resistance, where reproductive actions of insulin persist in ovaries, contrary to insulin-resistant tissues in the presence of abundant insulin levels. Such a paradox could be explained by metabolic, but not mitogenic, insulin signaling pathway defects [1]. Hyperinsulinemia in PCOS directly raises the level of LH and, therefore, indirectly enhances the secretion of androgen-mediated by LH. Moreover, hyperinsulinemia could suppress the synthesis of SHBG in the liver and cause the increase of free T. Not all PCOS subjects express hyperandrogenemia and/or hyperinsulinemia, so it is important to distinguish different PCOS phenotypes and propose targeted and individual therapeutic approaches for PCOS treatment.

Dyslipidemia frequently follows PCOS patients [65]. Our study showed PCOS-related increased levels of total cholesterol, triglycerides, and LDL cholesterol, as well as others [1], while after 4 weeks of administration, both kinds of treatment significantly reduced these parameters. However, HDL cholesterol levels did not change significantly, although we could speculate that SAE treatment alone seems to be the best candidate, tending to elevate HDL levels in the S + A group. It was shown that cyanidin-3-O-β-glucoside, orally administered to mice, was found to markedly increase the serum HDL levels [66], while the *Aronia melanocarpa* functional beverage was found to increase HDL in aging rats in the presence of herbs and pectins [67]. On the other hand, MET was able to restore levels of total cholesterol, triglycerides, and LDL cholesterol in the letrozole-induced PCOS rat model [68], as in our study.

Underlying all mentioned reproductive and metabolic changes observed in our study, OS alterations could provide one mechanistic basis for the opposite results obtained by applied protocols. It was known that OS could be considered one of the two main processes associated with PCOS pathogenesis, besides silent inflammation. On the other hand, the antioxidative properties of *Aronia melanocarpa* are largely evaluated and confirmed in different investigations and MET still represents the gold standard in PCOS treatment despite its well-known adverse effects. Here, we analyzed pro-oxidative parameters and antioxidative defense system in systemic circulation and ovarian tissue homogenates. Generally, markers of lipid peroxidation are displayed as TBARS and MDA levels in the blood and other tissues. In our study, TBARS levels were increased in systemic circulation and ovarian tissue in the P group, which was in accordance with others [68]. However, all three types of treatment significantly decreased TBARS levels. The dose of MET applied in our study (500 mg/kg) was able to prevent further lipid peroxidation in plasma, and ovarian tissue, contrary to a lower dose of MET (20 mg/kg) applied in similar PCOS investigations [41]. Moreover, one more study evaluated the effects of MET (300 mg/kg) in the pre-pubertal PCOS model, which was also unable to reverse MDA levels when applied alone, aside from in combination with resveratrol [26]. Therefore, we assumed that the dose applied in our study was sufficient to combat the formation of lipid peroxides and further free radical chain reactions of polyunsaturated fatty acids of the fatty acid membrane. Moreover, SAE exerted a similar effect as MET in preventing lipid peroxidation in both plasma and ovaries. PCOS-associated insulin resistance, hyperandrogenism, dyslipidemia, and obesity are also accompanied by increased lipid peroxidation [69], while SAE, as well as MET, showed integrated effects on mentioned perturbances. Pro-oxidative generators, such as O_2_^−^ and H_2_O_2_, showed similar oscillations in plasma, while levels of NO2- remained unaltered between groups. More interesting was what happened with the antioxidative defense system in two investigated compartments. Antioxidative cell machinery is important because SOD converts O_2_^−^ to H_2_O_2_, while H_2_O_2_ is converted to water molecules by the activity of CAT and glutathione peroxidase, while GSH serves as an electron donor in such reactions. The activity of CAT was decreased in the P group, but only SAE combined with MET was capable of improving levels of this antioxidant enzyme toward the control levels in blood and ovaries. However, SAE alone improved CAT activity in plasma also, contrary to the absent effect of MET alone treatment. Regarding SOD activity, there was a clear decrease in activity after PCOS induction into ovarian tissue, which was improved after the application of SAE, MET, and their combination. That result, regarding MET treatment applied in the same dose used in our study (500 mg/kg), could be compared with others [70]. However, when analyzing plasma SOD, PCOS decrement was abolished only in the MET alone treated group. Interestingly, SAE and SAE combined with MET did not significantly alter SOD levels compared to the P group. Altogether, regarding plasma antioxidative enzymes, we could conclude that the MET alone treatment did not alter CAT activity, while SAE (alone or in combination with MET) did not alter SOD activity compared to the P group. On the other hand, SAE (alone or in combination with MET) could reverse CAT, while those treatments failed to reverse SOD activity in comparison to the P group. GSH levels decreased in the P group and increased in SAE, MET, and combined groups in both plasma and ovaries.

## 5. Conclusions

Our findings strongly confirmed that SAE alone or in combination with MET was able to ameliorate reproductive and metabolic disturbances in the DHEA-induced PCOS rat model. SAE alone did not alter the protective effects of MET in PCOS, while there was a confirmed synergistic effect of these two agents in most of the estimated PCOS features. These results could serve as a base for further clinical investigations regarding SAE administration in PCOS women to evaluate the particular contribution of this extract on reproductive and metabolic profiles in this patient population, especially in those with obese PCOS phenotype.

## Figures and Tables

**Table 1:** Serum hormone levels.

Group	Testosterone	Estradiol	Progesterone
C	0.14 ± 0.05	78.78 ± 8.76	24.56 ± 7.40
P	2.95 ± 0.49 ^a^	118.00 ± 17.93	11.74 ± 1.02 ^A^
P + M	0.12 ± 0.04 ^b^	64.78 ± 7.88 ^B^	28.15 ± 6.72 ^B^
P + A	0.13 ± 0.05 ^b^	53.38 ± 7.84 ^B^	27.63 ± 6.83 ^b^
P + M + A	0.15 ± 0.09 ^b^	61.50 ± 11.03 ^B^	27.17 ± 80 ^B^

Levels of testosterone, estradiol, and progesterone; Data are presented as mean ± SEM. ^A^ Statistical significance at the level of *p* < 0.05 compared to P group, ^a^ Statistical significance at the level of *p* < 0.01 compared to C group, ^B^ Statistical significance at the level of *p* < 0.05 compared to P group, ^b^ Statistical significance at the level of *p* < 0.01 compared to P group (6 rats per group).

**Table 2:** Serum lipid profile in rats.

Group	Cholesterol	Triglycerides	HDL	LDL
C	2.07 ± 0.19	0.46 ± 0.09	0.77 ± 0.03	1.18 ± 0.07
P	2.82 ± 0.07 ^a^	1.05 ± 0.10 ^a^	0.68 ± 0.09	1.67 ± 0.05 ^a^
P + M	1.88 ± 0.20 ^b^	0.53 ± 0.12 ^b^	0.75 ± 0.12	1.00 ± 0.16 ^b^
P + A	1.92 ± 0.27 ^B^	0.50 ± 0.12 ^b^	0.95 ± 0.06	1.03 ± 0.11 ^b^
P + M + A	2.18 ± 0.21 ^b^	0.43 ± 0.11 ^b^	0.90 ± 0.08	0.14 ± 0.04 ^b^

Levels of total cholesterol, triglycerides, high-density lipoproteins, and low-density lipoproteins. Data are presented as mean ± SEM. ^a^ Statistical significance at the level of *p* < 0.01 compared to C group, ^B^ Statistical significance at the level of *p* < 0.05 compared to P group, ^b^ Statistical significance at the level of *p* < 0.01 compared to P group (6 rats per group).

## Data Availability

Data is contained within the article.
